# Robotic upper urinary tract reconstruction for ureteral stricture: a single-center series

**DOI:** 10.1007/s11701-025-02754-y

**Published:** 2025-09-07

**Authors:** Alice Bourillon, Lucas Freton, Juliette Hascoet, Claire Richard, Camille Haudebert, Gregory Verhoest, Romain Mathieu, Lee C. Zhao, Karim Bensalah, Benoit Peyronnet

**Affiliations:** 1 Department of Urology, Rennes University Hospital, Rennes, France; 2 New York University Langone Medical Center, New York, NY USA

**Keywords:** Robotics, Ureter, Stricture, Reconstruction, Treatment

## Abstract

The surgical approach of ureteral stricture has changed dramatically over the past 15 years with the rise of robotic upper urinary tract reconstruction. This study aimed to evaluate the outcomes of all robotic ureteral reconstructions performed at a single academic center for ureteral stricture and to assess the predictive factors of stricture recurrence. The charts of all patients who underwent robot-assisted ureteral reconstruction between 2013 and 2024 at a single academic center were retrospectively reviewed. Many different surgical techniques were used including non-refluxing reimplantation with or without psoas hitch, refluxing ureteral reimplantation (side-to-side), Boari flap, uretero-ureterostomy, ureterolysis and buccal mucosa graft (BMG) ureteroplasty. The primary outcome was the absence of stricture recurrence, defined as no need for repeat surgery, urinary drainage, or symptomatic upper urinary tract dilation at the last follow-up. Sixty patients, accounting for 63 ureteral reconstructions, were included in the final analysis. Twenty-five patients experienced early postoperative complications (40%), the majority being Clavien-Dindo grade 2. There were only three (5%) major complications (Clavien-Dindo grade 3b). Stricture recurrence occurred in six patients (10%), with a median time to recurrence of 2.5 months postoperatively. Radiotherapy was the only factor significantly associated with an increased risk of stricture recurrence. The present series confirm the overall low morbidity and low recurrence rate of robotic ureteral reconstruction using a variety of surgical techniques. Comparative studies with longer follow-up periods are necessary to evaluate outcomes in comparison to traditional surgical approaches.

## Introduction

Ureteral stricture is defined as a narrowing of the ureteral lumen which can result in obstruction of the urine flow and all the possible inherent complications such as obstructive pyelonephritis and impaired kidney function. Ureteral strictures are primarily iatrogenic, resulting mostly from abdominal, pelvic or endoscopic surgery [1]. The ureter’s anatomical characteristics—its concealed, tortuous retroperitoneal course and its fragile blood supply—make it particularly susceptible to injury.

Ureteral stricture repair remains a relatively unfamiliar procedure for many urologists. International guidelines primarily focus on oncologic and traumatic strictures, offering only limited detail on surgical techniques and lacking comprehensive decision-making algorithms [2]. Historically, the gold standard techniques have included open or laparoscopic ureteral reimplantation for short and/or distal ureteral stricture and autotransplantation and ileal ureteral substitution for longer and/or proximal strictures. However, these approaches are associated with significant complications, such as metabolic disturbances, recurrent urinary tract infections, and even renal function deterioration [3, 4].

Robotic surgery is now widely used in urology, with particularly evident benefits in reconstructive procedures. Since the first robotic ureteral reimplantation reported in 2003, the use of robot-assisted laparoscopic reconstruction has continued to grow steadily [5].

Robotic assistance has simplified established techniques, leading to reported reductions in operative time [6, 7] and has facilitated the development of novel approaches such as buccal mucosa graft ureteroplasty (BMG) and side-to-side reimplantation. However, the literature on robotic upper urinary tract reconstruction remains limited, with few studies and reviews available.

This study aimed to evaluate the outcomes of all robotic ureteral reconstructions performed at a single academic center for ureteral stricture and to assess the predictive factors of stricture recurrence.

## Material and methods

### Study design

The charts of all adult patients who underwent robotic upper urinary tract reconstruction for ureteral strictures between 2013 and 2024 at a single academic center were retrospectively reviewed and considered for inclusion. Cases involving ureteropelvic junction obstruction, kidney transplants, and ureteroileal strictures were excluded, as these represent distinct conditions with different therapeutic management. To focus exclusively on robot-assisted surgeries, open and laparoscopic procedures were also excluded. All remaining patients were included. The data collected encompassed patient age, gender, Body Mass Index (BMI), past medical history, prior radiotherapy or previous treatments for the stricture, characteristics of the stricture (length, location), preoperative assessment, duration of ureteral rest (defined as the number of days with no double-J stent prior to the surgical repair), use of indocyanine green, intraoperative ureteroscopy.

This study was conducted in accordance with the Declaration of Helsinki on ethical principles for medical research. This study was approved by the CNIL (Comité National Informatique et Liberté) under the reference number 2235966.

No funds, grants, or other support was received.

### Surgical techniques

All procedures began with docking the DaVinci Si, X or Xi system, with patient positioning and trocar placement adjusted according to the location of the stricture. The patient was placed in a flank position for proximal ureteral strictures and in a 20° to 30° Trendelenburg with spread legs for iliac and pelvic ureter strictures. Four robotic ports were used according to the standard set-up for robotic upper urinary tract surgery and robotic pelvic surgery, respectively. One 12-mm assistant port was used, and an extra 5-mm assistant port was added on a case-by-case basis. The DaVinci Si, X and Xi were used in all cases in the present series with a transperitoneal approach.

While intraoperative ureteroscopy and intravenous indocyanine green were never used at the beginning of the inclusion period, these tools were used routinely within the past years. Intraoperative ureteroscopy was used to guide the ureteral dissection with the ureteroscope introduced either through the nephrostomy tract in an antegrade fashion of through the urethra in a retrograde fashion. The stricture was located thanks to the ureteroscopy and IV indocyanine green was given in several cases to check the blood supply and excise the poorly vascularized tissue. The ureter was always meticulously dissected free from surrounding tissues, minimizing coagulation to preserve its vascular supply. In recent years, only the anterior aspect of the ureter was dissected first to avoid circumferential dissection and the inherent risk of compromising the blood supply if a BMG onlay was to be selected.

The purpose of this study was to evaluate the outcomes of robotic reconstruction without emphasizing a single specific procedure. Accordingly, eight different surgical techniques were employed: ureteral reimplantation for pelvic ureter strictures; psoas hitch, Boari flap, or buccal mucosa graft (BMG) for iliac ureter strictures; and ureteroureterostomy or BMG for lumbar ureter strictures. All patients were informed of the available options and provided consent for the chosen procedure. Six consultant surgeons were involved over the study period, but the most experienced surgeons were involved in 80% of the procedures.

The final surgical approach was determined intraoperatively at the surgeon’s discretion. The type of suture used was documented for all procedures.

An international expert was also involved by training two surgeons of our team and supervising the first few of buccal mucosa graft ureteroplasty remotely (telementoring). The most challenging indications were also discussed with this international expert during a yearly meeting for the first four years.

### Ureterolysis and intraperitonization

This technique was employed when ureteral dissection was deemed sufficient without the need for additional reconstruction. The ureter was carefully freed from surrounding inflammatory and fibrotic tissues without further surgical intervention. Intraoperative ureteroscopy was always used in those cases to ensure the lack of any persistent obstruction after ureterolysis.

### Ureteroureterostomy

This technique was selected for short strictures less than 2 cm in length, where a tension-free anastomosis could be achieved without the need for grafts or additional tissue mobilization. The two ureteral ends were spatulated on their 2 cm distal portion prior to perform the anastomosis.

### Ureteroneocystostomy

**Fig. 1:**
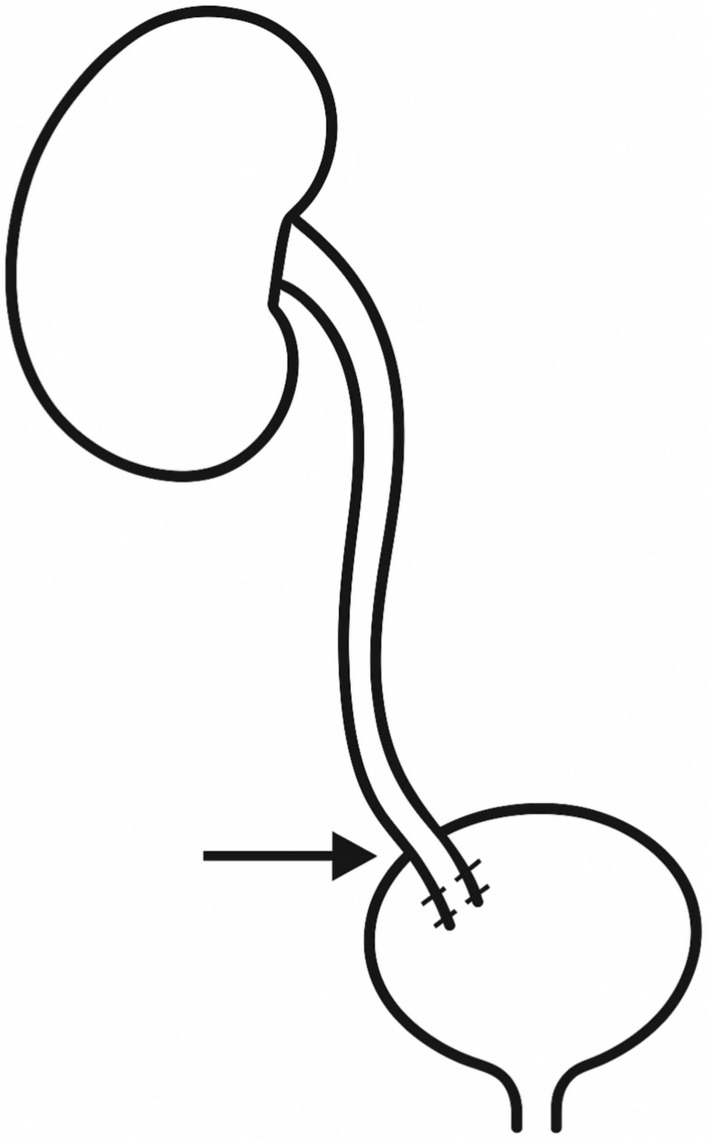
Schematic representation of a direct ureterovesical reimplantation

**Fig. 2:**
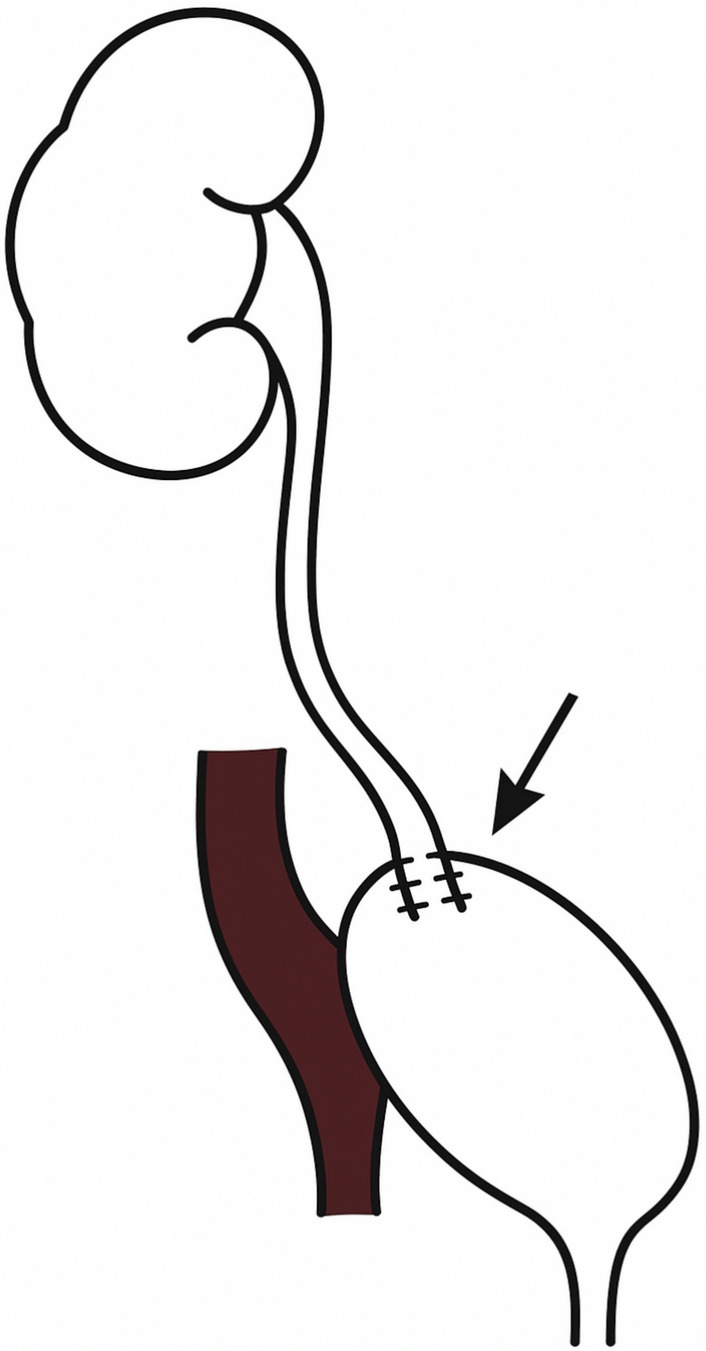
Schematic representation of a psoas hitch

Ureteral reimplantation (also known as ureteroneocystostomy) into the bladder was performed for distal strictures. The use of an antireflux technique was documented whenever performed. When an antireflux mechanism was created, it was always a Lich-Gregoir with opening of the detrusor on 4 cm and opening of the bladder mucosa only on the distal 1 cm of the detrusorotomy. The ureter was spatulated and its distal end was anastomosed to the bladder mucosa (Fig. 1). The detrusor was then closed on top of the distal ureter using interrupted Vicryl 2/0 sutures. When necessary, a psoas hitch was performed to ensure a tension-free anastomosis by mobilizing and securing the bladder to the psoas muscle with a V Lock 3/0, to spread the tension gradually (Fig. 2). Care was taken to place the sutures longitudinal to the psoas muscle and not too deep, to minimize the risk of nerve injury.

### Boari flap

This flap was used as an alternative when the psoas hitch did not provide sufficient mobilization. The bladder was mobilized and secured to the psoas muscle as previously described. The distal ureteral segment was excised to ensure healthy margins for the anastomosis. A transverse incision was then made in the bladder wall and closed longitudinally to create a tunnel-shaped flap. The anastomosis was subsequently performed between the distal ureter and the apex of this flap in a refluxing manner.

### Side-to-side reimplantation

**Fig. 3:**
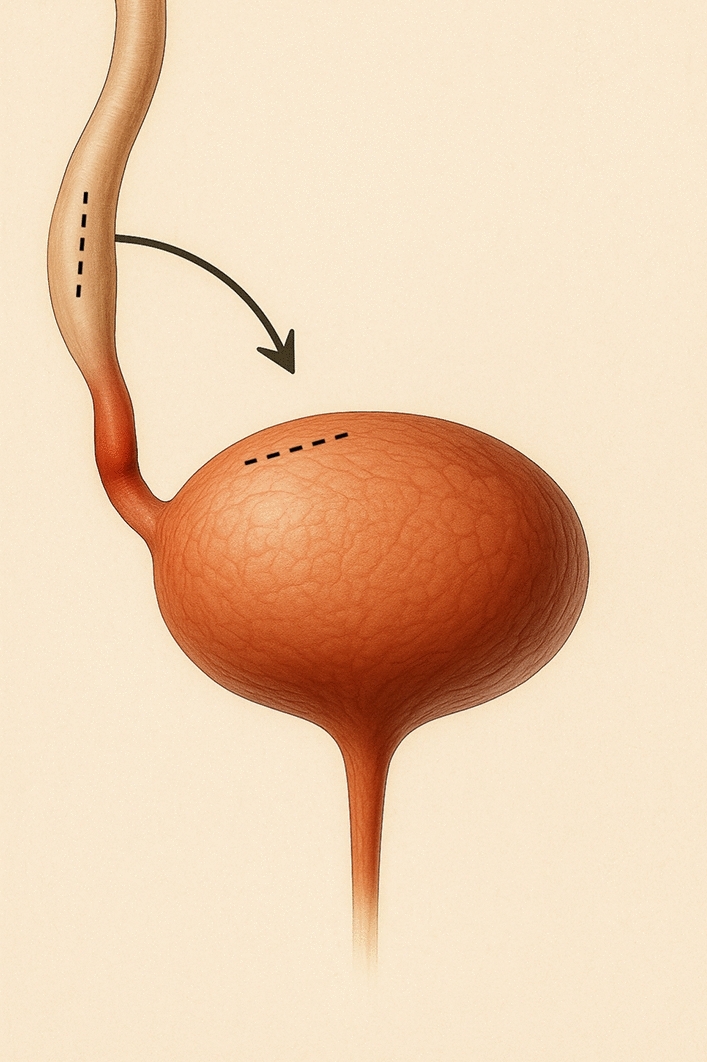
Schematic representation of a side-to-side reimplantation

Side-to-side reimplantation was another option for distal ureteral strictures. In this technique, both the bladder wall and the ureter were incised on their lateral aspects to avoid transecting the ureter. The ureter was then directly anastomosed to the bladder without creating an antireflux mechanism. This technique was elected in comorbid patients or difficult surgical environment to decrease the operative time and try to optimize the ureteral vascularization by avoiding transection (Fig. 3).

### Buccal mucosa graft ureteroplasty

The use of a buccal mucosa graft was another preferred option when the stricture exceeded 2 cm. A buccal mucosa patch was harvested and then anastomosed to the stricture site using running sutures after opening of the anterior fibrotic ureteral wall. If the posterior ureteral wall was also fibrotic, it was excised, and an augmented onlay patch repair was performed. An onlay anastomosis was feasible only when the ureter was not completely excised.

**Fig. 4:**
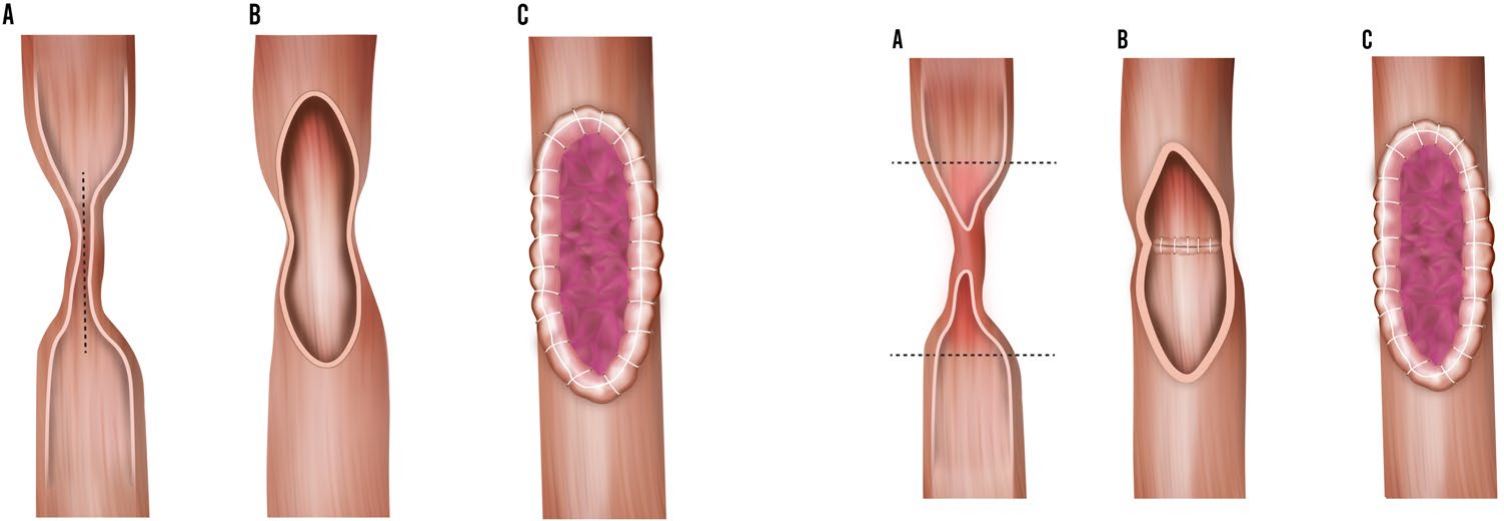
Schematic representation of buccal mucosa graft ureteroplasty: onaly (**a**) and augmented (**b**)

In all cases of BMG ureteroplasty, a fatty flap was brought and secured to the BMG to provide blood supply and a structural support, but also to provided tissue interposition on the anastomosis in case of fistula. An omental flap was used in most cases, but when not available or difficult to harvest, a peritoneal flap or a perirenal fat flap was used instead (Fig. 4).

### Outcomes of interest

The primary outcome was the stricture recurrence rate. Given the lack of a standardized definition in the literature, a composite criterion was established, including any of the following events: need for urinary drainage, reoperation, or symptomatic upper urinary tract dilatation observed at last follow-up. A stricture was considered recurrent if any of these conditions was met. The secondary outcomes of interest were the occurrence of early postoperative complications, defined as any complication occurring within 30 days following surgery, graded according to the Clavien–Dindo classification, and all the main perioperative outcomes (operative time, length of hospital stay,…) [8].

### Statistical analysis

Means and standard deviations were reported for continuous variables, medians and ranges for categorical variables and proportions for nominal variables. Comparisons between groups were performed using the χ^2^ test or Fisher’s exact test for discrete variables and the Mann–Whitney test for continuous variables as appropriate. The paired student t-test was used to evaluate the evolution of variables over time. Multiple comparisons were performed using analysis of variance (ANOVA) with a Bonferroni correction. Univariate logistic regression analyzes were performed to assess the risk factors of stricture recurrence. Statistical analyses were performed using JMP pro v.18.0.1 software (SAS Institute Inc., Cary, NC, USA). All tests were two-sided with *p* < 0.05 as a threshold to define statistical significance.

## Results

### Patients’ characteristics

Sixty patients were included in the study. Their characteristics are summarized in Table 1. Three patients underwent two surgeries due to stricture recurrence; thus, their demographic data were accounted for only once, but each stricture and recurrence were treated as separate events. The median age at surgery was 49 years (range 25–82), and the median BMI was 25 (range 17–42.3). Most patients had a history of prior pelvic surgery (N = 43, 72%). The most common presenting symptom was lumbar pain (N = 23, 38%), followed by asymptomatic cases found incidentally (N = 19, 32%). Seven patients (12%) had received previous radiotherapy. The median stricture length was 3 cm, with most of the strictures located in the pelvic ureter (N = 42, 67%). Twenty-four patients had failed a previous endoscopic treatment of the stricture (38%) and six had failed an attempt of surgical repair (10%). Most patients had undergone at least one previous pelvic surgical procedure (72%).

**Table 1:** Patients’ characteristics

	N = 60
*Gender*	
Female	43 (72%)
Male	17 (28%)
Median age (range)	49 (25–82)
Median BMI (range)^a^	25 (17–42.3)
Previous pelvic surgery, n (%)	43 (72%)
Smoker ^a^	7 (12%)
Previous radiotherapy, n (%)	7 (12%)
*Stricture cause*^b^	
Surgery	31 (49%)
Ureteroscopy	11 (17%)
Radiotherapy	6 (10%)
Others	16 (25%)
*Stricture location*^c^	
Lumbar	10 (16%)
Iliac	11 (17%)
Pelvic	42 (67%)
Ureteropelvic junction	1 (2%)
Previous attempt of endoscopic treatment of the stricture	24 (38%)
Previous attempt of surgical repair of the stricture	6 (10%)
Median stricture length, cm (range)	3 (0.5–10)

^a^Data was missing in four patients

^b^Four patients had both surgical and radiotherapic causes

^c^Two patients had iliac and pelvic stricture, and one had lumbar, iliac and pelvic stenosis. The ureteropelvic junction stricture was attributed to repeated ureteroscopies rather than to ureteropelvic junction syndrome and was therefore included

### Surgical techniques and perioperative management

As presented in Table 2, considering the 63 strictures treated, the surgical techniques employed were as follows: 17 psoas hitch procedures, 14 BMG ureteroplasties, 15 ureteroneocystostomies, 6 Boari flaps, 5 ureteroureterostomies, 4 side-to-side reimplantations, 2 ureterolysis. The most used suture for anastomosis was PDS 5/0, employed in 19 patients (30%). All but one patient received a postoperative double-J stent (98%). The double-J stent was removed prior to the reconstruction in 47 patients (75%) with a median duration of ureteral rest of 100 days (range: 1–300). Intraoperative ureteroscopy was used in 18 cases (29%) and IV indocyanine green was used in 35 cases (56%). Most of the ureteroneocystostomies were non-refluxing (87%). The median operative time was 195 min, with a median blood loss of 50 mL.

**Table 2:** Surgical techniques and perioperative management

	N = 63
*Technique*	
BMG	14 (22%)
Boari flap	6 (10%)
Side-to-side reimplantation	4 (6%)
Ureteroneocystostomy with anti-reflux system	13 (21%)
Ureteroneocystostomy without anti-reflux system	2 (3%)
Psoas hitch	17 (27%)
Ureteroureterostomy	5 (8%)
Ureterolysis	2 (3%)
Indocyanine green use^a^	35 (56%)
Intraoperative ureteroscopy^b^	18 (29%)
Postoperative ureteral stenting	62 (98%)
Median blood loss (range, cc)^c^	50 (0–600)
Ureteral rest	47 (75%)
Median operative time (range, min)d	195 (70–395)
Median duration of ureteral rest (days, range)^c^	100 (1–300)

^a^Data was missing for two procedures

^b^Data was missing for one procedure

^c^Data was missing for 16 procedures

^d^Data was missing for 14 procedures

### Perioperative outcomes

The postoperative outcomes are summarized in Table 3. The median hospital stay was 3 days, the median duration of urethral catheter drainage was 7 days, and the double-J stent was removed at a median of 6 weeks postoperatively. One patient required prolonged urinary catheter drainage due to the occurrence of macroscopic hematuria.Twenty-five patients experienced early postoperative complications (40%), the majority being Clavien-Dindo grade 2 (N = 15, 60% of all complications). There were only three (5%) major complications (Clavien-Dindo grade 3b): Two patients needed re-stenting due to obstructive pyelonephritis at the time of ureteral stent removal, and one patient needed emergency reoperation at day one after an ureteral reimplantation because of a rupture of the ureterovesical anastomosis.

**Table 3:** Post-operative results

	N = 63
Median length of hospital stay (range, days)	3 (1–10)
Median urethral catheter drainage, (range, days)^a^	7 (0–42)
Median ureteral stent drainage, (range, weeks)^a^	6 (3–15)
Early postoperative complications	25 (40%)
*Clavien grade*	
1	7 (28%)
2	15 (60%)
3b	3 (12%)
4 or 5	0 (0%)
Early redo surgery	2 (3%)
Symptomatic stricture recurrence at last follow-up	6 (10%)

^a^Data was missing for one patient

### Stricture recurrence

The median follow-up duration was 10 months. The estimated glomerular filtration rate remained stable between the preoperative work-up (mean: 82 ml/min) and last postoperative follow-up (mean: 80.5 ml/min; p = 0.52). No symptomatic reflux was observed during follow-up. At the last follow-up, 6 patients (10%) experienced symptomatic stricture recurrence, diagnosed due to lumbar pain, with imaging-confirmed upper urinary tract dilatation on abdominal CT scan, and underwent successful redo surgeries: 2 initial psoas hitches, 2 BMG, and 2 Boari flaps. Strictures were observed at nearly all anatomical levels (four pelvic, one iliac, and one lumbar). To maintain clinical consistency, redo surgeries were deemed successful based on the same primary outcome as the initial procedures—that is, the absence of the need for drainage, additional surgery, or symptomatic upper urinary tract dilatation at the last follow-up.Previous radiotherapy was the only factor significantly associated with recurrence (OR = 10.4, p = 0.02, see Table 4). Among the eight cases performed in irradiated patients there was three recurrences vs. three out of 55 non-radiated cases (38% vs. 5%; p = 0.02). All ureteral strictures occurring after radiotherapy were located in the distal ureter. While failures in the overall cohort occurred at proximal, mid, and distal sites, no significant difference in success rates according to stricture location was observed. The stricture recurrence rate did not differ significantly between BMG ureteroplasty, ureteral reimplantation and other techniques (14.3% vs. 5.7% vs. 14.3%; p = 0.51). Moreover, the use of ICG or intraoperative ureteroscopy did not significantly affect the rate of stricture recurrence (p = 0.22 and 0.6, respectively).

**Table 4:** Predictive factors of stricture recurrence in univariate regression analysis

Variable	Odds Ratio	CI95%	p-value
Ureteral rest	1.78	0.26–35.67	0.59
Gender (female vs male)	1.95	0.28–3.89	0.53
Previous failed surgical repair	2.08	0.10–16.88	0.56
Previous failed endoscopic treatment	1.71	0.29–10	0.53
Intraoperative ureteroscopy	1.25	0.16–7.08	0.81
Use of IV indocyanine green	0.24	0.02–1.61	0.16
Body Mass Index	1.05	0.90–1.20	0.49
Age	0.97	0.91–1.13	0.45
History of radiotherapy	10.4	1.58–17.82	0.02*
Stricture length	1.39	0.91–2.21	0.12
*Stricture location*			
Pelvic	1 (Ref)		
Iliac	1	0.04–27.98	0.99
Lumbar	0.92	0.12–19.20	0.94
*Surgical technique*			
Ureteral reimplantation	1 (Ref)		
BMG ureteroplasty	2.75	0.30–25.08	0.34
Other	2.75	0.30–25.08	0.34

## Discussion

Strictures of the upper urinary tract may be asymptomatic but can also lead to lumbar pain, recurrent urinary tract infections, and eventually kidney failure. Moreover, patients with ureteral strictures often report a reduced quality of life compared to the general population [9, 10]. Ureteral strictures can be managed using various techniques and surgical approaches. In recent years, robotic upper tract reconstruction has expanded significantly and is now regarded as the approach of choice in many centers [11–13]. However, the data on this approach are relatively scant and limited to a handful of highly experienced tertiary referral center which may question their reproducibility. In the present single center series, we observed that robotic upper tract reconstruction yielded satisfactory outcomes with a low morbidity and recurrence rate.

The introduction of robotic surgery into routine clinical practice marked a significant breakthrough in the early 2000s—particularly in urology [14]. Robotic assistance offers numerous advantages, including high-definition 3D magnified vision (enhanced when combined with fluorescence imaging), improved instrument dexterity, and greater ergonomic comfort for the surgeon. Its adaptability and relatively short learning curve make it a highly effective alternative to traditional laparoscopic and open surgical approaches. Robotic platforms also enabled the development of innovative techniques, such as buccal mucosa graft (BMG) ureteroplasty, side-to-side reimplantation or appendiceal flap ureteroplasty [15–17]. Our results confirm the promising outcomes reported by others for robotic upper urinary tract reconstruction [1, 18]. Although it was not possible to prove is statistically, we believe that the many surgical principles and optimized perioperative management including a thorough preoperative work-up, the use of intraoperative ureteroscopy, intravenous indocyanine green, ureteral rest preoperatively and ureteral stenting postoperatively contributed, along with the aforementioned assets of robotic surgery, to the successful outcomes observed in many cases in this series.

The volume/experience could also play a role. Ureteral stricture repair is seen as common general urology surgical procedures. However, they remain relatively uncommon and are often spread across many different centers and surgeons. Our center is recognized as a tertiary referral center, mostly because one of the senior surgeon has been supervising the majority of the procedure in the present series (50 out of 63) and has been performing about the same number at outside institutions. However, the prevalence of robotic ureteral reconstruction in other French centers has not been reported and one should acknowledge that there is no consensual definition for a high volume center.

Received an increasing number of patients over the past ten years, with one surgeon being involved in most of the procedures, which may have played a role to soften the learning curve and improved the overall outcome reported herein.

Another important finding of our series is the relatively low rates of complications observed, and especially the low rate of major postoperative complications, despite a relatively challenging population with the vast majority of patients having undergone previous pelvic surgical procedures. No patient in our series died postoperatively nor had to go to the intensive care unit. The only serious complications that were observed were one urinary fistula and two early recurrences of the stricture. This is in line with the existing literature which underscores the very rare occurrence of life-threatening complications after ureteral reconstruction [19]. We believe this is another strong incentive to offer ureteral reconstruction to all ureteral strictures patients fit for surgery rather than leaving them with indwelling ureteral stents or nephrostomy tubes which severely affect their quality of life and are associated with high rates of complications in the long term [20].

While a recent series has raised awareness on the issue of post reimplant reflux, none of the patients in the present series who underwent side to side reimplant reported symptomatic reflux, which can be a potential complication following reimplantation without an anti-reflux mechanism—particularly in side-to-side anastomoses, which were performed in four patients in this series. Had symptomatic reflux occurred, the authors would have considered a new procedure incorporating an anti-reflux system, such as the Lich-Gregoir or Politano-Leadbetter techniques.

The only predictive factor of recurrence was an history of radiotherapy with a recurrence rate of 38% in this population. Radiation therapy is known to alter the urothelium and promote extracellular matrix remodeling and fibrosis of the whole urinary tract wall including the ureter [21]. Radiation therapy compromises the blood supply and overall hampers the healing process. Hence the increased risk of recurrence we observed is not surprising and in line with previous series [22]. This highlights the need for further research on this specific patients’ population to determine the best possible approach for ureteral stricture in radiated patients both in terms of surgical techniques and perioperative management (e.g. could there be a role for hyperbaric oxygen therapy?).

The present study has several limitations that should be acknowledged. First, it has all the biases inherent to its retrospective design. This is a non-comparative study, although a comparison with open surgery—which remains the gold standard until official guidelines evolve—would have been valuable. Certain preoperative information, such as precise stricture measurements, was occasionally unavailable; however, based on existing literature, this is unlikely to have had a significant impact on the overall outcomes [23].

Our primary outcome—a composite criterion including three clinical events—did not incorporate survival. We considered that a functional assessment based on pain and symptoms was more relevant in the context of ureteral strictures. Unlike several studies in the literature, we chose not to include renal dysfunction or isolated radiographic hydronephrosis in our main outcome measure. We found that isolated dilation, in the absence of symptoms or renal impairment, was of limited clinical relevance. Instead, we prioritized patient-centered factors with tangible real-life impact, such as pain and the need for reoperation. Additionally, the criteria guiding each surgeon’s choice of technique were not explicitly defined. Another limitation is the potential variability in surgical technique between operators, as multiple surgeons were involved. To mitigate this, we aimed to report as many procedural details as possible. The relatively short-term follow-up prevents to draw any robust conclusion on the long-term reliability of robotic upper urinary tract reconstruction for ureteral stricture. The lack of long-term follow-up is indeed regrettable, as late complications or recurrences may have occurred. Unfortunately, most patients were lost to follow-up after the first postoperative consultation, and no data were available beyond the early postoperative period. Furthermore, no detailed data were collected on the redo procedures which precluded to analyze the key fac tors contributing to the success of these reoperations which can be regarded as a shortcoming.

Finally, although this represents one of the largest series in the literature, the relatively small sample size still warrants caution.

## Conclusion

Robotic ureteral stricture repair is feasible using a variety of surgical techniques, with encouragingly low rates of recurrence and morbidity. Establishing clear guidelines is essential to support urologists in selecting the most appropriate procedure, as ureteral strictures can vary greatly in etiology and anatomical presentation, requiring tailored management strategies. Our findings suggest that previous radiotherapy may be associated with a higher risk of recurrence. Further prospective comparative studies are needed to confirm this association and refine patient selection criteria.

## Data Availability

No datasets were generated or analysed during the current study.
